# [Expression of Concern] Human ether-à-go-go-related gene mutation L539fs/47-hERG leads to cell apoptosis through the endoplasmic reticulum stress pathway

**DOI:** 10.3892/ijmm.2026.5975

**Published:** 2026-09-02

**Authors:** Shuting Ma, Yun Zhao, Miaomiao Cao, Chaofeng Sun

Int J Mol Med 43: 1253-1262, 2019; DOI: 10.3892/ijmm.2019.4049

Following the publication of the above paper, it has been drawn to the Editor's attention by an interested reader that, regarding the Hoechst-stained images shown in Fig. 4 on p. 1258, the 'WT-hERG+L539fs/47-hERG' panel (top row, on the right) and the 'L539fs/47-hERG+4-PBA' panel (bottom row, middle panel) shared an overlapping section, such that data which were intended to show the results of differently performed experiments had apparently been derived from the same original source. Furthermore, upon performing an independent analysis of the data in this paper in the Editorial Office, it came to light that the western blot data featured in Fig. 5A contained a number of potential anomalies: there appeared to be possible breaks in continuity in the gel showing the blots for cleaved caspase-3 in this figure, and the backgrounds for the western blots in this figure also appeared to be rather heterogenous.

Although the authors responded to this initial query concerning the incorrect assembly of data in Fig. 4, up to this time no response from them has been forthcoming concerning the western blots shown in Fig. 5. Owing to the time that has now elapsed, and given the fact that the Editorial Office has been made aware of potential problems associated with the scientific integrity of this study, we are issuing an Expression of Concern to notify readers of these issues while the Editorial Office continues to investigate this matter further.

